# Immunotoxicity of Silver Nanoparticles (AgNPs) on the Leukocytes of Common Bottlenose Dolphins (*Tursiops truncatus*)

**DOI:** 10.1038/s41598-018-23737-0

**Published:** 2018-04-04

**Authors:** Wen-Ta Li, Hui-Wen Chang, Wei-Cheng Yang, Chieh Lo, Lei-Ya Wang, Victor Fei Pang, Meng-Hsien Chen, Chian-Ren Jeng

**Affiliations:** 1 Graduate Institute of Molecular and Comparative Pathobiology, National Taiwan University, Taipei, 10617 Taiwan; 2 College of Veterinary Medicine, National Chiayi University, Chiayi, 60004 Taiwan; 3 Farglory Ocean Park, Hualien, 97449 Taiwan; 4 Department of Oceanography and Asia-Pacific Ocean Research Center, National Sun Yat-sen University, Kaohsiung, 80424 Taiwan

## Abstract

Silver nanoparticles (AgNPs) have been extensively used and are considered as an emerging contaminant in the ocean. The environmental contamination of AgNPs is expected to increase greatly over time, and cetaceans, as the top ocean predators, will suffer the negative impacts of AgNPs. In the present study, we investigate the immunotoxicity of AgNPs on the leukocytes of cetaceans using several methods, including cytomorphology, cytotoxicity, and functional activity assays. The results reveal that 20 nm Citrate-AgNPs (C-AgNP_20_) induce different cytomorphological alterations and intracellular distributions in cetacean polymorphonuclear cells (cPMNs) and peripheral blood mononuclear cells (cPBMCs). At high concentrations of C-AgNP_20_ (10 and 50 μg/ml), the time- and dose-dependent cytotoxicity in cPMNs and cPBMCs involving apoptosis is demonstrated. C-AgNP_20_ at sub-lethal doses (0.1 and 1 μg/ml) negatively affect the functional activities of cPMNs (phagocytosis and respiratory burst) and cPBMCs (proliferative activity). The current study presents the first evidence of the cytotoxicity and immunotoxicity of AgNPs on the leukocytes of cetaceans and improves our understanding of environmental safety concerning AgNPs. The dose-response data of AgNPs on the leukocytes of cetaceans are invaluable for evaluating the adverse health effects in cetaceans and for proposing a conservation plan for marine mammals.

## Introduction

Silver nanoparticles (AgNPs) have been extensively used in numerous commercial products including textiles, cosmetics, and health care items mainly due to their strong antimicrobial properties^1,2^. Previous studies estimated that the production of AgNPs and the number of AgNP-containing products would increase over time^3,4^. Furthermore, AgNPs can be released during the production, transport, use, and/or disposal of AgNP-containing products, subsequently draining into the aquatic environment and ultimately accumulating in the ocean^5,6^. Although the fate of AgNPs in the aquatic environment is complicated and changeful, previous studies have indicated that AgNPs in the aquatic environment can remain as individual particles in suspension, aggregate, dissolve, react with different species in the environment, or be regenerated from Ag^+^ ions^2,3^. Therefore, the extensive use and growing production of AgNP-containing products may aggravate the environmental contamination level of AgNPs, leading to concerns about the safety and environmental toxicity of AgNPs and related ecotoxicological investigations of AgNPs in the marine environment.

AgNPs may precipitate in marine sediments, be ingested by benthic organisms, and thereby enter the food chain in the marine environment^7^. The current knowledge on ecotoxicological data regarding AgNPs in the marine ecosystem is still scarce, and only limited data on the potential toxicity of Ag-NPs to marine organisms at different trophic levels has been reported^7–12^. Their results have demonstrated that AgNPs are toxic to all tested marine organisms in a dose-dependent manner, suggesting that AgNPs may have negative effects on marine organisms at different trophic levels within the marine ecosystem. In addition, AgNPs can be transferred from one trophic level to the next via the food chain and may have negative effects on the animals at higher trophic levels, such as marine mammals^5,7,8^. The environmental contamination level of AgNPs is expected to increase greatly in the near future^3,4^, and marine mammals, as the top predators in the ocean, will suffer the potentially negative impacts caused by AgNPs.

Although no studies on the toxicity of AgNPs in marine mammals have been reported, AgNPs evidently induce several negative effects, such as hepatitis, bile duct hyperplasia, nephritis, neuron cell apoptosis, and alteration of gene expression of the brain, in laboratory mammals^13–18^. Previous *in vitro* studies using different cell lines have demonstrated that AgNPs can damage DNA, cell membranes, and mitochondria through reactive oxygen species (ROS) dependent/independent pathways and thereby induce cell apoptosis and necrosis^19,20^. Previous studies conducted in laboratory mammals including mice and rats have also demonstrated that AgNPs can enter the blood circulation through oral and inhalation exposure, and then accumulate in multiple organs^13,16,18,21–23^. Several studies on the effects of AgNPs in human leukocytes have been recently reported^24–32^. These studies demonstrated that AgNPs could cause several effects in human neutrophils, including morphological alterations, cytotoxicity, atypical cell death, inhibition of de novo protein synthesis, increased production of the CXCL8 chemokine (IL-8), and impaired lysosomal activity^28–31^. Cytotoxicity and inhibition of lymphocyte proliferation in lymphocytes and macrophages were also revealed^24–26,32^. However, the effects of AgNPs on the functional activities of human neutrophils and lymphocytes are still poorly understood. Therefore, the negative effects of AgNPs on the white blood cells of marine mammals such as cetaceans are of concern.

Despite such concern, no studies on the toxicity of AgNPs in cetaceans have been reported. Generally, *in vivo* studies are usually not feasible, and ethical issues concerning the study of immunotoxic effects caused by environmental contaminants in cetaceans are difficult to overcome, so *in vitro* study using blood samples from captive cetaceans would be a logical and crucial approach^33^. Therefore, we evaluate the effects of AgNPs on the leukocytes of cetaceans by several methods, including cytomorphological examination (microscopic examination under Liu’s stain/autometallographic [AMG] Ag visualization and transmission electron microscope [TEM]), cytotoxicity assays (flow cytometric analysis using Annexin V/Propidium iodide staining), and functional activity assays (ROS production, phagocytosis, and respiratory burst of polymorphonuclear cells [PMNs] and proliferative activity of peripheral blood mononuclear cells [PBMCs] by flow cytometry). Furthermore, previous studies have demonstrated that the toxicity and physicochemical characteristics of AgNPs are associated with their surface coating and size^19,20^. The state of AgNPs and Ag in the marine environment and in the bodies of cetaceans is complicated and various, and it is not realistic to investigate all possible types of AgNPs and Ag compounds in the leukocytes of cetaceans *in vitro*. Considering the wide usage of 20 nm citrate-AgNPs (C-AgNP_20_) in recently reported studies of human leukocytes^26,29,30^, commercial C-AgNP_20_ is used in the present study.

## Results

### Characterization of C-AgNP_20_

**Figure 1:**
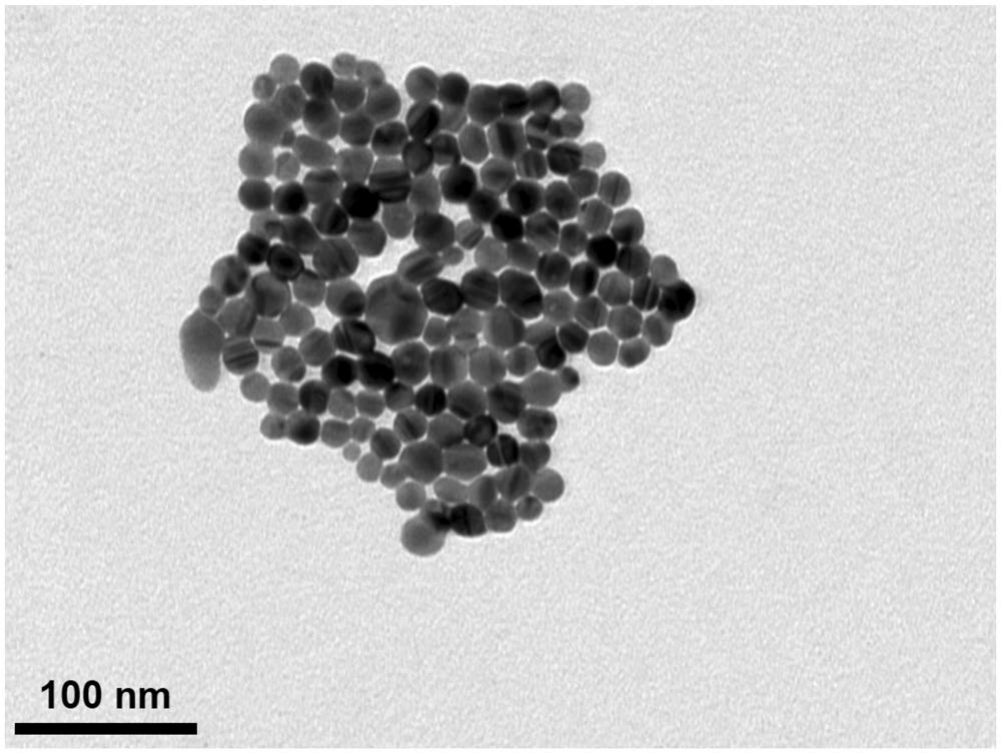
Characterization of C-AgNP_20_. The representative TEM image of C-AgNP_20_ in complete RPMI-1640.

The C-AgNP_20_ suspended in complete RPMI-1640 medium (RPMI-1640 [Gibco, NY, USA] with 10% fetal bovine serum, 2mM L-glutamine, 50 IU penicillin, and 50 µg streptomycin) were spherically shaped and close to 20 nm in diameter (Fig. 1). The size distributions were 30 ± 0 (100%) and 30 ± 0 (100%) for C-AgNP_20_ at 100 and 500 μg/ml, respectively. The values of the zeta potentials were −38.97 ± 1.33 and −44.2 ± 1.35 mV for C-AgNP_20_ at 100 and 500 μg/ml. The Poly-dispersity Indexes (PDIs) were 0.12 ± 0.00 and 0.11 ± 0.01, indicating that the distribution consisted of a single size mode without aggregates (The detailed information is presented in Supplementary Files).

### C-AgNP_20_ induced different cytomorphological alterations and intracellular distributions in cetacean PMNs/PBMCs

**Figure 2:**
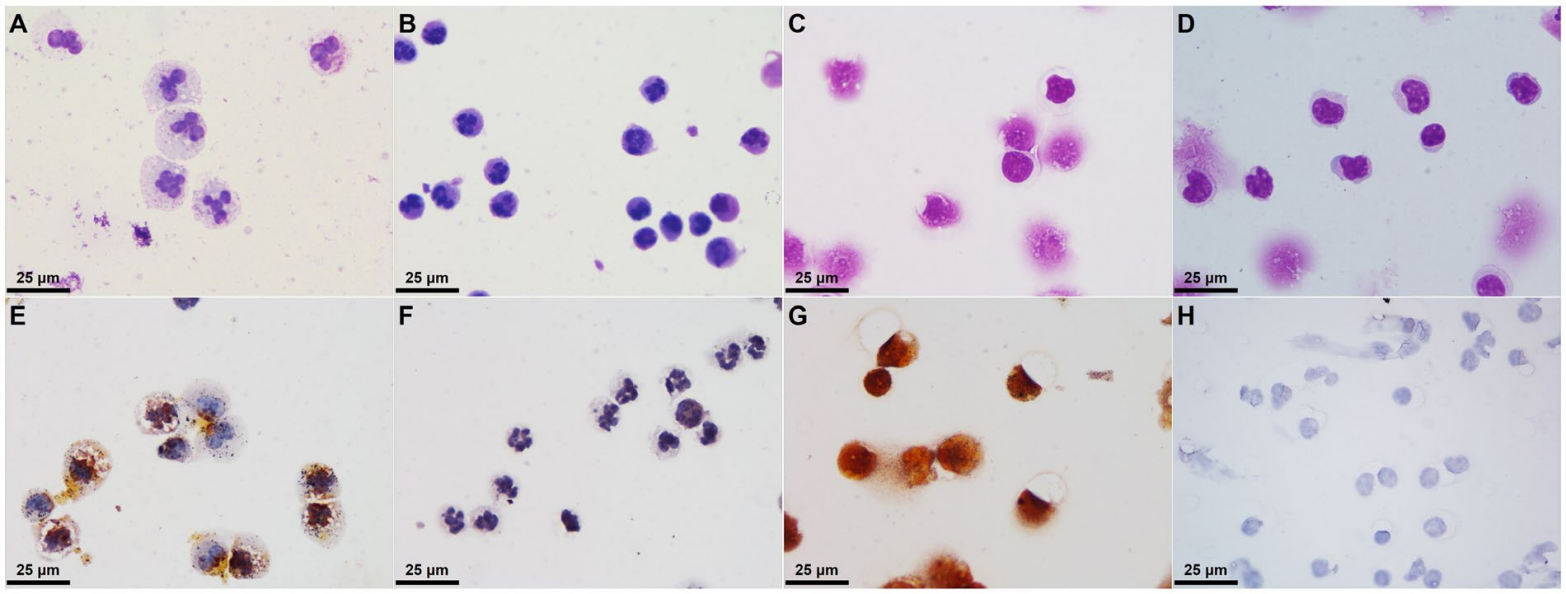
C-AgNP_20_ induced different cytomorphological alterations and intracellular distributions in cPMNs and cPBMCs. Representative cytological slides stained with Liu’s stain of cPMNs after 4 h of culture (**A**) with 50 μg/ml C-AgNP_20_ and (**B**) without C-AgNP_20_ (control), cPBMCs after 4 h of culture (**C**) with 50 μg/ml C-AgNP_20_ and (**D**) without C-AgNP_20_ (control); representative cytological slides stained with silver enhancement method of cPMNs after 4 h of culture (**E**) with 50 μg/ml C-AgNP_20_ and (**F**) without C-AgNP_20_ (control); cPBMCs after 4 h of culture (**G**) with 50 μg/ml C-AgNP_20_ and (**H**) without C-AgNP_20_ (control).

**Figure 3:**
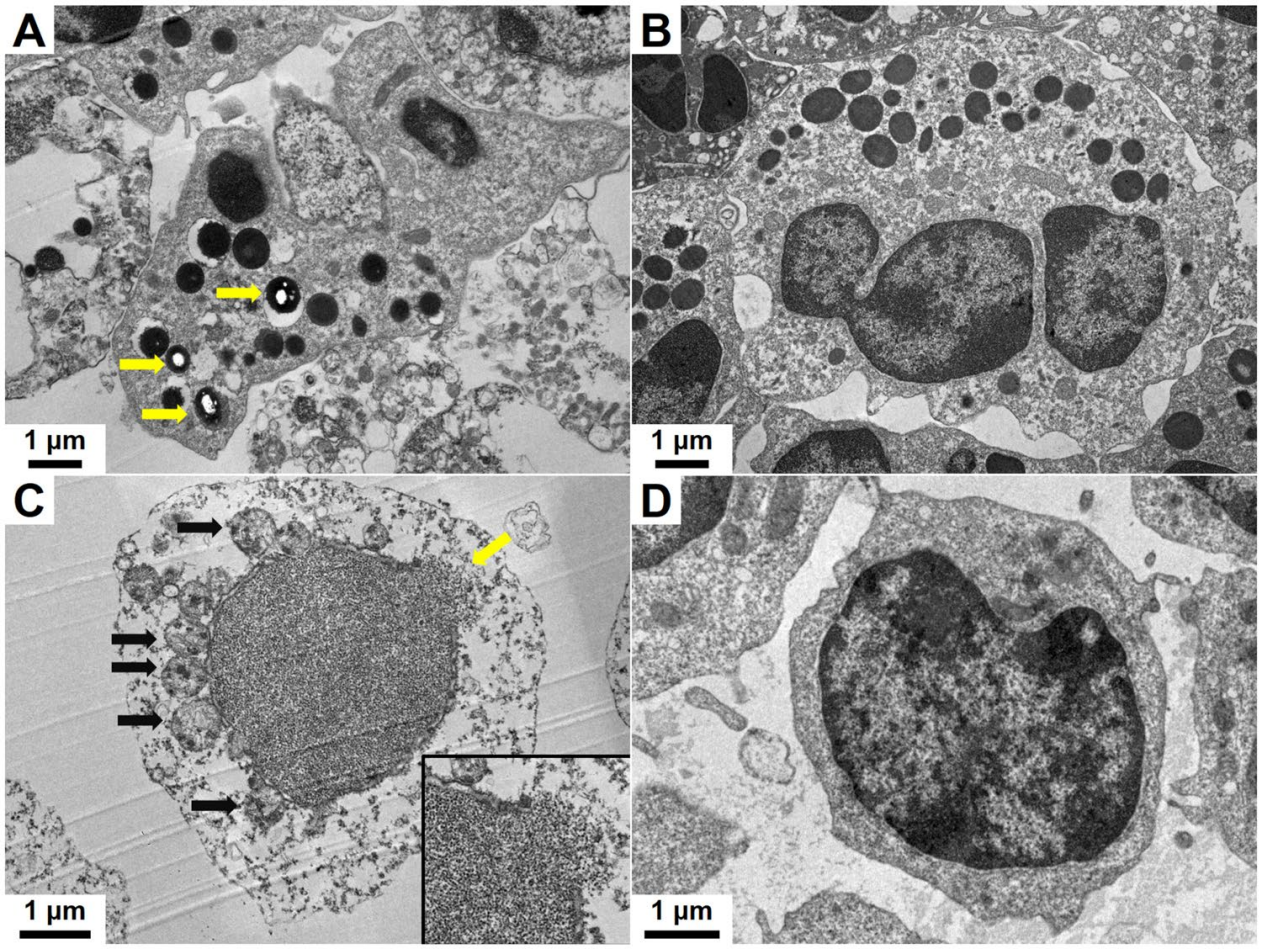
C-AgNP_20_ induced different cytomorphological alterations and intracellular distributions in cPMNs and cPBMCs. Representative TEM images of ultrathin sections of cPMNs after 4 h of culture (**A**) with 50 μg/ml C-AgNP_20_ and (**B**) without C-AgNP_20_ (control). Intracellular electron-dense structures with clear spaces are indicated by yellow arrows. Representative TEM images of ultrathin sections of cPBMCs after 4 h of culture (**C**) with 50 μg/ml C-AgNP_20_ and (**D**) without C-AgNP_20_ (control). Ruptured nuclear membrane is indicated by yellow arrow, and black arrows indicate the swollen mitochondria with cristae disruption. Inset: higher power field of the ruptured nuclear membrane.

The cell size of cetacean PMNs (cPMNs) with 50 μg/ml C-AgNP_20_ treatment was increased by the presence of variably sized intracytoplasmic vacuoles and enlarged multi-lobed nuclei (Fig. 2A), which were not observed in the control (Fig. 2B). The numbers of cetacean PBMCs (cPBMCs) with blurred cell morphology increased after exposure to 50 μg/ml C-AgNP_20_. Although the presences of intracytoplasmic vacuoles were found in the cPBMCs with 50 μg/ml C-AgNP_20_ treatment, the increased cell size of cPBMCs was unremarkable (Fig. 2C) as compared to the control (Fig. 2D). The AMG positive signals in the cPMNs were mainly intracytoplasmic black dots with the multifocal presence of an amorphous golden yellow to brown substance in the cytoplasm and nucleus (Fig. 2E). The AMG positive signals in the cPBMCs were an intracellularly diffuse, golden yellow to brown substance (Fig. 2G). No AMG positive signals were found in the controls (Fig. 2F and H). Under TEM, intracellular electron-dense structures with clear spaces were found in the intracytoplasmic vacuoles of some but not all cPMNs with 50 μg/ml C-AgNP_20_ treatment for 4 h, but no particles of compatible size and shape with C-AgNP_20_ were noted (Fig. 3A). No intracellular electron-dense structures were found in the control group (Fig. 3B). Nuclear membrane rupture and swollen mitochondria with cristae disruption were frequently found in the cPBMCs with 50 μg/ml C-AgNP_20_ treatment for 4 h (Fig. 3C) but were not found in the control group (Fig. 3D).

**Figure 4:**
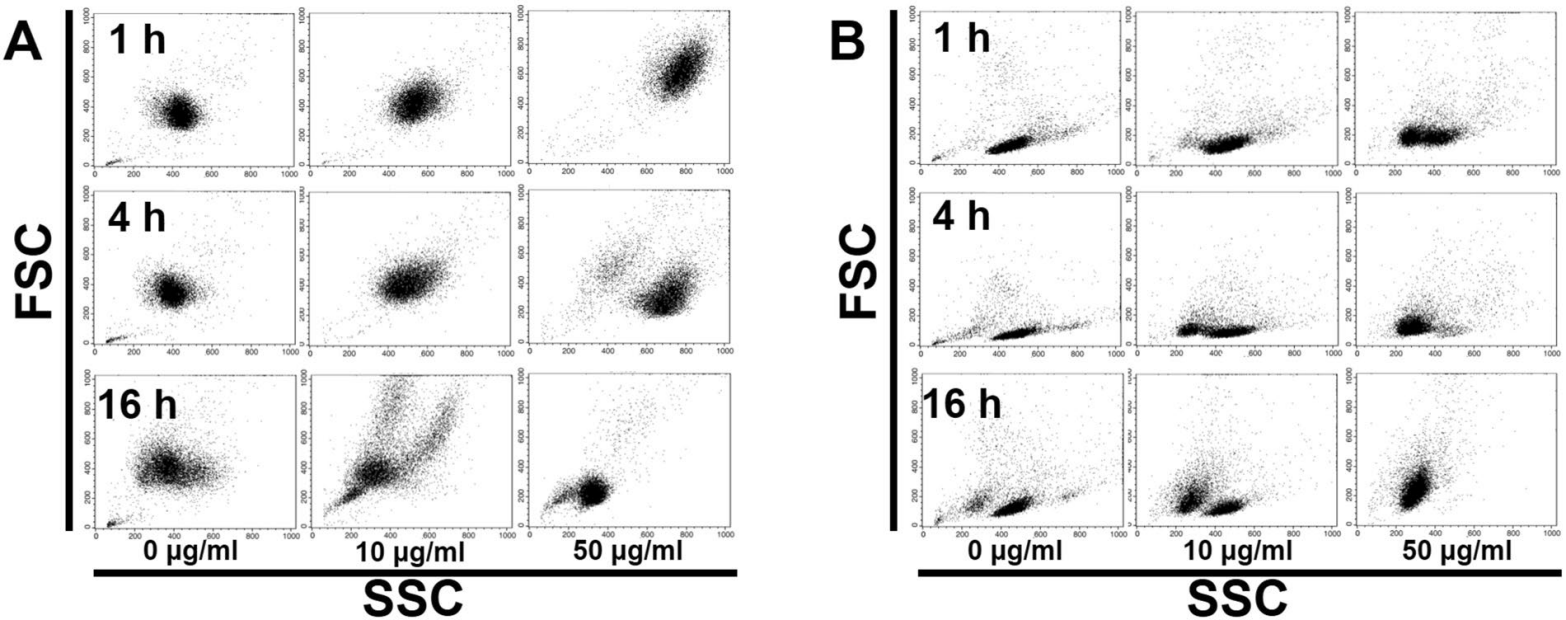
Representative dot plots by flow-cytometric analyses for evaluation of the cell size and inner complexity of (**A**) cPMNs and (**B**) cPBMCs.

In the flow-cytometric analysis, the inner complexity of cPMNs with 10 and 50 μg/ml C-AgNP_20_ increased after 1 and 4 h of culture. The cell size of cPMNs with 10 and 50 μg/ml C-AgNP_20_ increased after 1 h of culture but decreased after 4 h of culture. Decreased cell size with mildly increased inner complexity was found in cPBMCs after 1 and 4 h of culture with 10 and 50 μg/ml C-AgNP_20_. After 16 h of culture with 10 and 50 μg/ml C-AgNP_20_, both cPMNs and cPBMCs revealed decreased cell size and inner complexity. No marked differences were noted between the cPMN and cPBMC exposures to 0, 0.1 and 1.0 μg/ml C-AgNP_20_ after 1 and 4 h of culture (Fig. 4A and B).

### C-AgNP_20_ induced cytotoxicity in cPMNs and cPBMCs with involvement of apoptotic pathway

**Figure 5:**
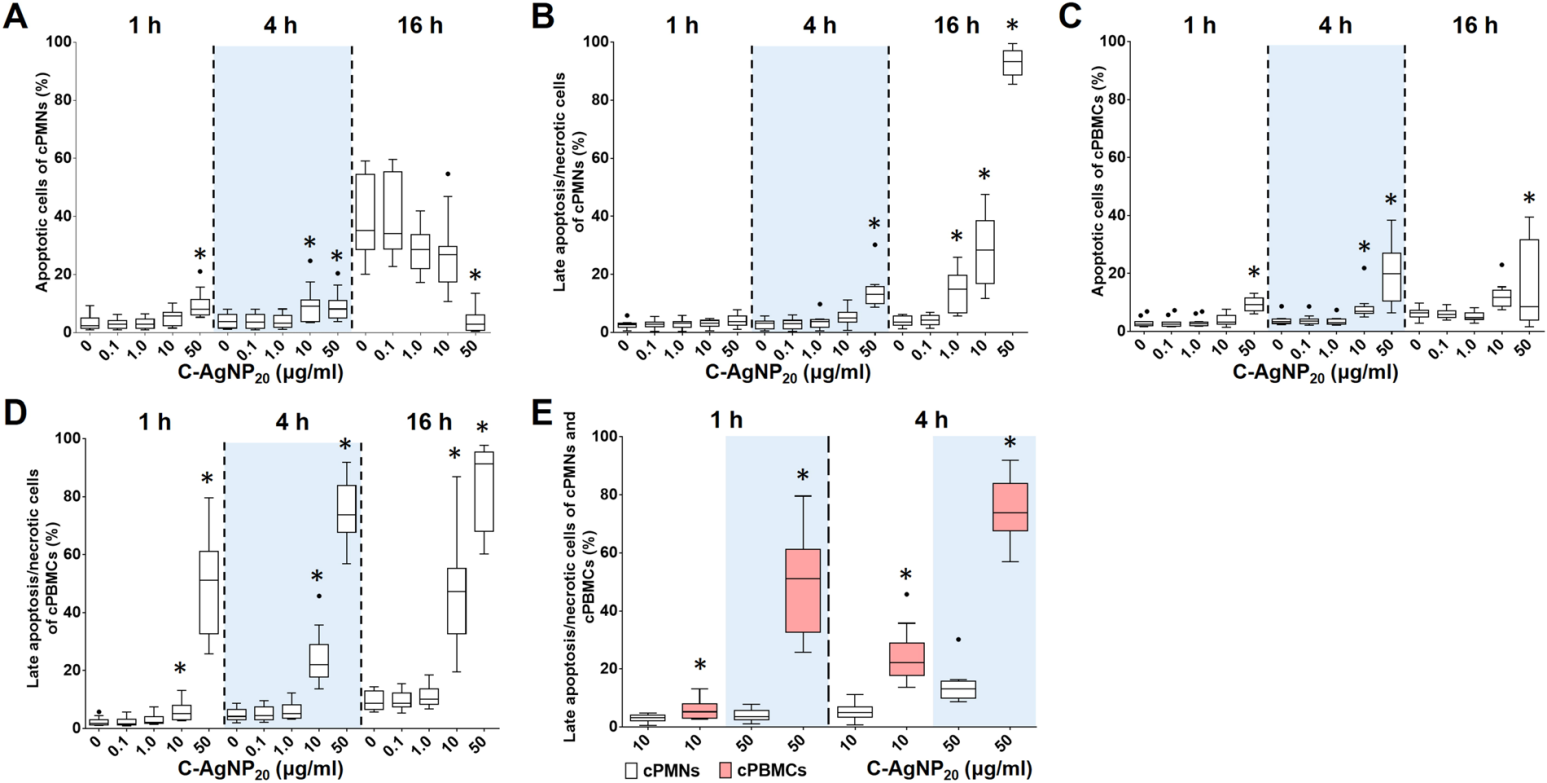
Cytotoxicity of C-AgNP_20_ in cPMNs and cPBMCs. Percentages of apoptotic and late apoptotic/necrotic cells of (**A** and **B**) cPMNs and (**B** and **C**) cPBMCs after exposure to different concentrations of C-AgNP_20_ and different incubation times. Comparison of the cytotoxicity caused by C-AgNP_20_ in cPMNs and cPBMCs (**E**). The bar in the middle of the box represents the median, and the bottom and top of the box describe the first and third quartiles. The whiskers show the 75^th^ percentile plus 1.5 times IQR and 25^th^ percentile minus 1.5 times IQR of all data, and any values greater than these are defined as outliers and plotted as individual points. Asterisks indicate statistically significant differences from the control (*p* < 0.05, Kruskal-Wallis Test for [A to D] and Mann-Whitney U-test for [E]).

C-AgNP_20_ (10 and 50 μg/ml) treatment significantly increased the number of apoptotic cells and late apoptotic/necrotic cells in a time- and dose-dependent manner in cPMNs and cPBMCs. Percentages of apoptotic and late apoptotic/necrotic cells of cPMNs and cPBMCs after exposure to different concentrations of C-AgNP_20_ and incubation time are shown in Fig. 5A to D. A significant increase in the apoptotic rate was observed in the cPMNs (median ± interquartile range (IQR): 8.04 ± 5.27%; p = 0.0003) and cPBMCs (9.32 ± 4.36%; p < 0.0001) after 1 h of 50 μg/ml C-AgNP_20_ treatment. Late apoptotic/necrotic cells in cPMNs (13.08 ± 5.92%; p < 0.0001) and cPBMCs (51.19 ± 28.52%; p < 0.0001) after 4 h and 1 h of 50 μg/ml C-AgNP_20_ treatments, respectively, were significantly increased. The rates of late apoptotic/necrotic cells in cPBMCs with 10 and 50 μg/ml C-AgNP_20_ after 1 and 4 h of culture were significantly higher than those of cPMNs with the same treatments (*p* < 0.0001; except the group with 10 μg/ml C-AgNP_20_ after 1 h of culture [*p* = 0.0232]) (Fig. 5E). A marked increase in the number of apoptotic cells was noted in cPMNs without C-AgNP_20_ after 16 h incubation, which might be similar to the spontaneous apoptosis found in human neutrophils^29,35^.

**Figure 6:**
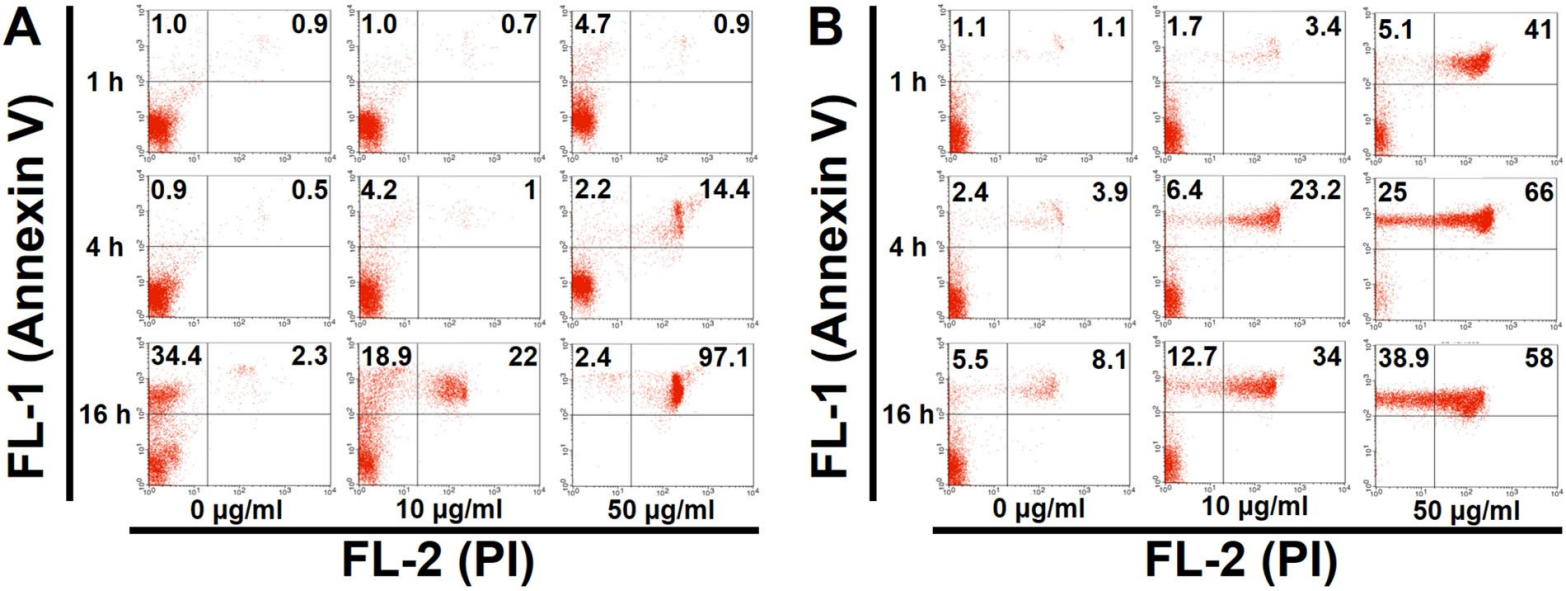
Representative dot plots by flow-cytometric analyses using Annexin V-FITC/PI double staining in (**A**) cPMNs and (**B**) cPBMCs exposed to different concentrations of C-AgNP_20_ and different incubation times.

There were no statistically significant differences in the percentages of apoptotic and late apoptotic/necrotic cells in cPMNs and cPBMCs with 0, 0.1, and 1.0 μg/ml C-AgNP_20_ after 1 and 4 h of culture. Based on the results of morphological alterations and cytotoxicity assays, 0.1 and 1.0 μg/ml C-AgNP_20_ were defined as the sub-lethal dose for cPMNs and cPBMCs. Representative dot plots by flow-cytometric analyses of cPMNs and cPBMCs exposed to 0, 10 and 50 μg/ml C-AgNP_20_ for different incubation times are shown in Fig. 6. The results illustrated that cPMNs and cPBMCs exposed to C-AgNP_20_ were apoptotic and subsequently shifted to late apoptotic/necrotic, suggesting the apoptotic pathway is involved in the cytotoxicity induced by C-AgNP_20_.

### C-AgNP_20_ at sub-lethal dose significantly decreased the phagocytosis but increased the respiratory burst in cPMNs

**Figure 7:**
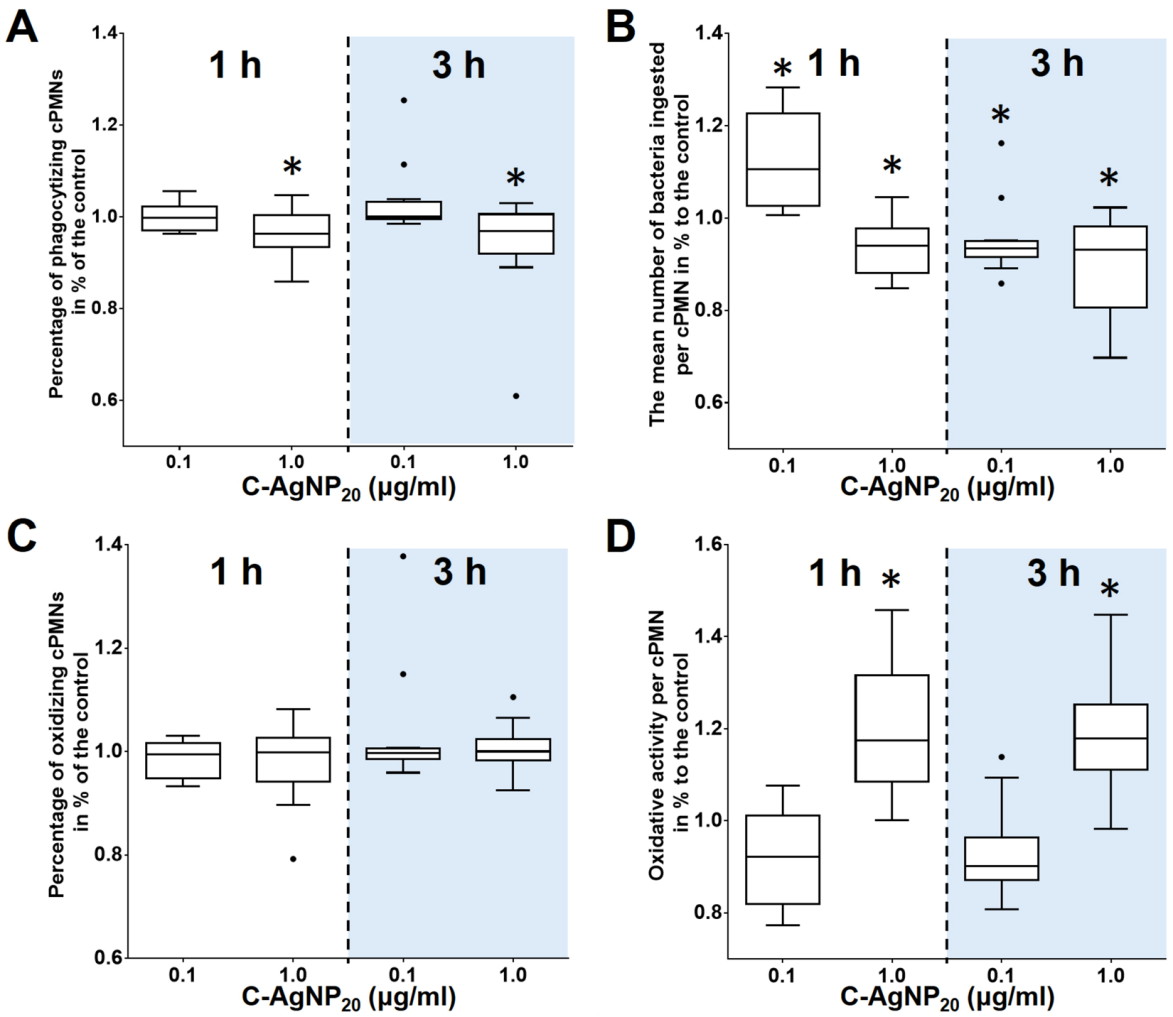
The negative effects of C-AgNP_20_ on the functional activity of cPMNs. (**A**) percentage of phagocytizing cPMNs, (**B**) number of ingested bacteria per cPMN, (**C**) percentage of oxidizing cPMNs, and (**D**) oxidative activity per cPMN. The bar in the middle of the box represents the median, and the bottom and top of the box describe the first and third quartiles. The whiskers showed the 75^th^ percentile plus 1.5 times IQR and 25^th^ percentile minus 1.5 times IQR of all data, and any values greater than these are defined as outliers and plotted as individual points. Asterisks indicate statistically significant differences from the control (*p* < 0.05, Kruskal-Wallis Test).

**Figure 8:**
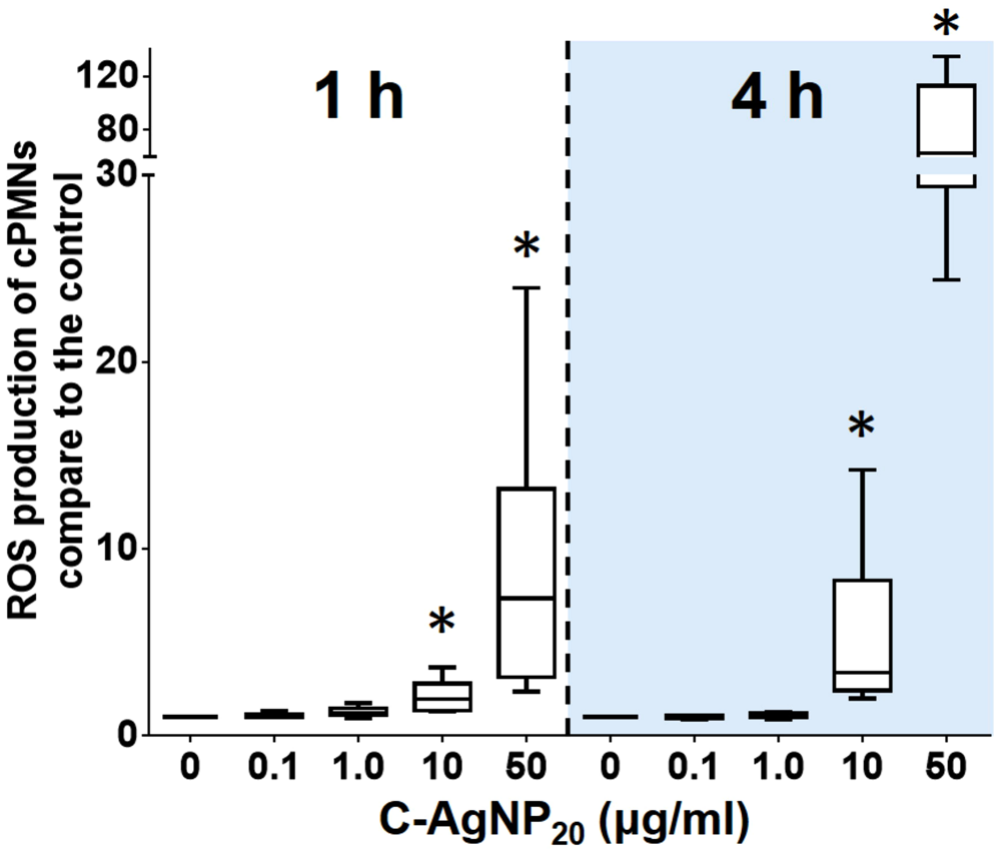
The ROS production of cPMNs induced by C-AgNP_20_. The bar in the middle of the box represents the median, and the bottom and top of the box describe the first and third quartiles. The whiskers show the 75^th^ percentile plus 1.5 times IQR and 25^th^ percentile minus 1.5 times IQR of all data, and any values greater than these are defined as outliers and plotted as individual points. Asterisks indicate statistically significant differences from the control (*p* < 0.05, Kruskal-Wallis Test).

The percentage of phagocytizing cPMNs was significantly decreased after 1 and 3 h of C-AgNP_20_ (1.0 μg/ml) treatment (Fig. 7A). The number of ingested bacteria per cPMN significantly increased after 1 h of C-AgNP_20_ (0.1 μg/ml) treatment, but significantly decreased after exposure to C-AgNP_20_ (0.1 μg/ml) for 3 h and C-AgNP_20_ (1.0 μg/ml) for 1 and 3 h (Fig. 7B). No significant difference in the percentages of oxidizing cPMNs was found after 1 and 3 h of exposure to C-AgNP_20_ (Fig. 7C), but C-AgNP_20_ (1.0 μg/ml) induced a significant increase in the oxidative activity per cPMN after 1 and 3 h of exposure (Fig. 7D). In order to determine whether the increased respiratory burst of cPMNs exposed to C-AgNP_20_ was associated with oxidative killing or spontaneous ROS production, the ROS production of cPMNs induced by C-AgNP_20_ without bacterial stimulation was performed. Our results indicated that the ROS production of cPMNs was significantly increased after exposure to 10 and 50 μg/ml C-AgNP_20_ for 1 and 4 h exposure, but no significant differences in ROS production were noted in the cPMNs exposed to 0, 0.1 and 1.0 μg/ml C-AgNP_20_ (Fig. 8).

### C-AgNP_20_ at sub-lethal dose significantly decreased proliferative activity of cPBMCs

**Figure 9:**
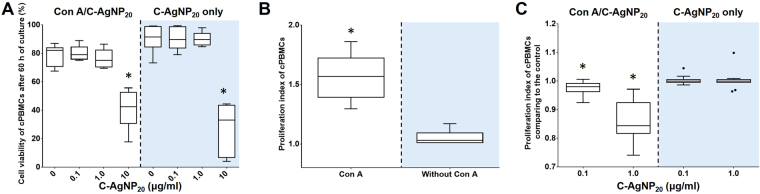
The negative effects of C-AgNP_20_ on the functional activity of cPBMCs. (**A**) The cell viability of cPBMCs exposed to C-AgNP_20_ with or without Con A stimulation after 60 h of culture. (**B**) Con A-induced proliferative activity in cPBMCs. (**C**) The effects of C-AgNP_20_ on the proliferative activity of cPBMCs. The bar in the middle of the box represents the median, and the bottom and top of the box describe the first and third quartiles. The whiskers show the 75^th^ percentile plus 1.5 times IQR and 25^th^ percentile minus 1.5 times IQR of all data, and any values greater than these are defined as outliers and plotted as individual points. Asterisks indicate statistically significant differences from the control (*p* < 0.05, Kruskal-Wallis Test for [A and C]; Mann-Whitney U-test for [B]).

C-AgNP_20_ (10 μg/ml) caused a significant decrease in cell viability of cPBMCs after 60 h of culture (with Concanavalin A [Con A]: Me: 42.45%, p = 0.0047; without Con A: Me: 33.15%, *p* = 0.005) (Fig. 9A). Therefore, 0.1 and 1.0 μg/ml C-AgNP_20_ were considered sub-lethal doses for the proliferative activity assay of cPBMCs. Con A significantly induced cell proliferative activity in cPBMCs in contrast to the cPBMCs without Con A (Fig. 9B). The Con A-induced cell proliferative activity of cPBMCs was significantly inhibited by 0.1 and 1.0 μg/ml C-AgNP_20_ treatment (Fig. 9C).

## Discussion

The present study has demonstrated the first evidence of the cytotoxicity and immunotoxicity caused by AgNPs on the leukocytes of cetaceans. Our data demonstrated that C-AgNP_20_ at high concentrations (10 and 50 μg/ml) induced a time- and dose-dependent cytotoxicity in cPMNs and cPBMCs, and an apoptotic pathway was involved in the C-AgNP_20_-induced cytotoxicity. The cPBMCs were more vulnerable than cPMNs to the C-AgNP_20_-induced cytotoxic effects, suggestive of a cell-type-specific response to AgNPs. Furthermore, the functional activities of cPMNs and cPBMCs were significantly compromised by C-AgNP_20_ at sub-lethal doses (0.1 and 1.0 μg/ml). The biodistribution of AgNPs or Ag in cetaceans is still largely unknown, but previous *in vivo* studies of AgNPs and Ag by oral exposure in laboratory rats demonstrated that the silver concentration is approximately 10 times higher in the liver than in blood or plasma^16,21,23^. Based on the concept of these laboratory animal models, it is presumed that the Ag concentration in the blood of stranded cetaceans may range from 0.01 to 72.6 μg/ml^34,35^. Although the state of Ag and AgNPs in the body of cetaceans is undetermined, complicated, and various, the current results still suggest that the immune function of cetaceans may have been compromised by AgNPs and/or Ag, and the immunotoxic effects of AgNPs in marine mammals should not be overlooked.

Two studies have used the same C-AgNP_20_ (Pelco^®^ citrate Biopure™ silver, Ted Pella, CA, USA) to investigate the effects of AgNPs on human PMNs^29,30^. The characterization of the C-AgNP_20_ has been studied, but the endotoxin level of C-AgNP_20_ has not been investigated^29,30^. Endotoxins are common contaminants in engineered nanomaterials and can influence the results of immunological and toxicological studies due to their marked immunostimulatory effects^36^. Therefore, the endotoxin levels of the C-AgNP_20_ used in the present study were investigated. These studies have demonstrated that human PMNs are well tolerant to C-AgNP_20_, and only an increased apoptotic rate is observed after 24 h of culture with 100 μg/ml C-AgNP_20_ treatment^29,30^. Therefore, the lethal dose of C-AgNP_20_ in human PMNs is considered to be 100 μg/ml. In contrast, our data indicated that the lethal dose of C-AgNP_20_ in cPMNs is ≥10 μg/ml; hence, it might suggest that C-AgNP_20_ cause different cytotoxic effects on cPMNs and human PMNs.

In the current study, C-AgNP_20_ (10 and 50 μg/ml) significantly induced cytotoxicity in cPBMCs. This finding is similar to those of studies conducted in human lymphocytes with C-AgNP_20_ treatment^26,27^. In addition, a cell-type-specific response of human PBMCs exposed to polyvinylpyrrolidone (PVP)-coated AgNPs (55 to 90 nm) has been demonstrated and may be associated with differences in cell-type-specific uptake and intracellular distribution of AgNPs between monocytes and lymphocytes^25^. Our data on cytotoxicity assays found that the cytotoxicity induced by C-AgNP_20_ was significantly higher in cPBMCs than in cPMNs, which might be associated with differences in cell-type-specific uptake and intracellular distribution. The intracellular distribution of AgNPs in human neutrophils in a previous study indicated that C-AgNP_20_ could penetrate into neutrophils and localize in intracytoplasmic vacuoles within 1 min^29^. In contrast, the intracellular distribution of AgNPs in lymphocytes is still undetermined. A previous study found that uncoated AgNPs treatment (75 to 130 nm) could induce varying degrees of cell deformity, cell membrane damage, and vacuolization in human lymphocytes, but no AgNPs or agglomerated AgNPs were identified in lymphocytes under TEM^24^. It is speculated that larger-sized AgNPs (75 to 130 nm) might not be able to penetrate the cell membrane and may only be uptaken by phagocytosis or endocytosis. In contrast, smaller-sized AgNPs might directly penetrate the cell membrane, and thus the interaction between smaller-sized AgNPs and cPBMCs might be different from that in the previous study using larger-sized AgNPs.

The results of TEM in our study demonstrated that C-AgNP_20_ agglomerated in the intracytoplasmic vacuoles of cPMNs, compatible with the results of AMG visualization and a previous study conducted in human neutrophils^29^. In addition, the presence of amorphous AMG positive signals in the cytoplasm and nucleus of cPMNs suggested that the C-AgNP_20_ were partially dissolved, released Ag^+^ ions, and caused cytotoxicity^37,38^. Therefore, although C-AgNP_20_ were sequestered in the intracytoplasmic vacuoles of cPMNs, they could still induce certain negative effects due to the intracellular dissolution. Conversely, diffuse AMG positive signals found in cPBMCs suggested that the C-AgNP_20_ might be diffusely dispersed in the cytoplasm and nuclei of cPBMCs. However, no particles of C-AgNP_20_ were found under TEM. As above, our results suggest that the C-AgNP_20_ directly penetrated into the cytoplasm and nuclei of cPBMCs, intracellularly dissolved (Trojan-horse type mechanism), and thereby induced a stronger cytotoxicity in cPBMCs than in cPMNs. Furthermore, it is also suggested that the cytotoxicity of C-AgNP_20_ on cPMNs and cPBMCs may be associated with the particles themselves and the released Ag^+^ ions.

ROS are strongly bactericidal but may also cause damage to the cell itself, driving the cell into apoptosis^39^. A previous study using citrate-coated AgNPs (20 and 70 nm) found that AgNPs did not significantly increase ROS production in human PMNs^30^. Nevertheless, other studies using PVP-coated AgNPs (10 nm and 50 nm) and uncoated AgNPs (15 nm) demonstrated that AgNPs could increase the level of ROS production and thereby induce ROS-dependent cytotoxicity in human PMNs^28,31^. In the current study, the increased ROS production was observed only in the cPMNs with 10 and 50 μg/ml C-AgNP_20_ treatment, and significant cytotoxicity was observed in the cPMNs with the same treatments. Therefore, our data suggested that C-AgNP_20_ may cause cytotoxicity in cPMNs by a ROS-dependent pathway.

To determine the effects of C-AgNP_20_ on the functional activity in cPMNs and cPBMCs, sub-lethal doses (0.1 and 1.0 μg/ml) of C-AgNP_20_ were used in functional activity assays for both cPMNs and cPBMCs. Our data demonstrated that C-AgNP_20_ at sub-lethal doses could suppress the phagocytosis but stimulate the respiratory burst of cPMNs. A previous study using human PMNs found that the phagocytosis and the respiratory burst were not affected by 10 μM (approximately 1 μg/mL AgNPs [2.0 nm to 34.7 nm in diameter]) after 15 min of incubation^40^. Although the type and size of AgNPs and the protocol in the previous study are different from those in our study, it is still suggested that AgNPs can differently affect the functional activities of PMNs in different animal species. To our knowledge, this is the first study demonstrating that AgNPs can cause suppressive effects on the phagocytosis of PMNs, so the underlying mechanism is still poorly studied. It is speculated that the suppressive phagocytosis may be associated with the restricted expansion capability and/or the impaired phagocytosis of PMNs^41–43^. In our study, C-AgNP_20_ were ingested by cPMNs and agglomerated in the intracytoplasmic vacuoles, and it is possible that the expansion capability of cPMNs may be restricted by the presence of intracytoplasmic vacuoles. However, no marked morphocytological alterations were noted on the cPMNs with sub-lethal doses of C-AgNP_20_, and thus the mechanism of suppressive phagocytosis cannot be solely explained by the restricted expansion capability. On the other hand, the process of phagocytosis in humans can be generally classified into 3 stages, (1) attachment of target particles, (2) pseudopod extensions around attached particles, and (3) engulfment of attached particles in a phagosome^43^, and defeat in any stage can result in impaired phagocytosis. Previous studies have indicated that contaminants such as heavy metals (mercury, aluminium, and cadmium), polychlorinated biphenyls, and perfluorinated compounds mostly cause a suppressive effect on phagocytosis of cPMNs^33,44–46^. However, the molecular mechanism of phagocytosis of PMNs in humans and animals is complicated and has not been well-studied, and the molecular mechanism and process of phagocytosis in cetaceans might be different from that in humans. Therefore, further experiments for expanding the knowledge on the molecular mechanism of phagocytosis of cPMNs are required to determine the interactions between AgNPs and phagocytosis of cPMNs.

Our data demonstrated that the respiratory burst of cPMNs was stimulated by C-AgNP_20_. Several possible mechanisms should be considered to explain the stimulated respiratory burst of cPMNs exposed to C-AgNP_20_. First, the increased respiratory burst of cPMNs might be associated with the spontaneous ROS production induced by C-AgNP_20_. However, the increased respiratory burst of cPMNs in the present study cannot be solely explained by the ROS production induced by C-AgNP_20_ because no significantly increased ROS production was observed in cPMNs exposed to 0.1 and 1.0 μg/ml C-AgNP_20_. Hence, it is speculated that the number of C-AgNP_20_ ingested by cPMNs increased during active phagocytosis. Bacteria with appropriate opsonisation are recognized by PMNs via specific surface receptors, and the phagocytosis of PMNs will be activated^42,43^. In addition, a previous study of interactions between AgNPs and bacteria demonstrated that AgNPs could accumulate in the membrane of bacteria, while some of them might penetrate into the bacteria^47^. Therefore, the bacteria might have been a vector for the C-AgNP_20_ in our study. Both phenomena could increase the number of C-AgNP_20_ ingested by cPMNs and thereby increase the ROS production of cPMNs. As above, our data suggest that the increased respiratory burst of cPMNs is associated with spontaneous ROS production induced by C-AgNP_20_ rather than the oxidative killing of cPMNs against bacteria.

The inhibitory effect caused by C-AgNP_20_ (0.1 and 1.0 μg/ml) on Con A-induced proliferative activity in cPBMCs was evident in our study. Previous studies have demonstrated that no inhibitory effects on the proliferative activity are noted in human PBMCs treated with <10 μg/ml uncoated AgNPs (1 to 2.5 nm and 75 to 130 nm in diameter), citrate-coated AgNPs (24.3 ± 4.5 nm), and PVP-coated AgNPs (75 ± 20 nm and 21.6 ± 4.8 nm in diameter)^24–26,32^. This difference suggests that C-AgNP_20_ can cause proliferative arrest of cPBMCs at relatively lower concentration (≤1 μg/ml), and further implies that cPBMCs are more vulnerable than are human PBMCs. The possible mechanisms on the proliferative arrest induced by C-AgNP_20_ in cPBMCs include cell cycle arrest, intracellular calcium transients, chromosomal aberrations, and cytoskeleton deformations^48–51^. The mechanism of AgNPs on the proliferative arrest in cPBMCs needs further investigation.

The current study presents the first evidence of the cytotoxicity and immunotoxicity of AgNPs on the leukocytes of cetaceans and improves our understanding of environmental safety concerning AgNPs. The dose-response data of AgNPs on the leukocytes of cetaceans are invaluable for evaluating the adverse health effects in cetaceans and for proposing a conservation plan for marine mammals. Furthermore, the suppressive effects on the phagocytosis of cPMNs caused by AgNPs, which were not found in previous human studies, indicate that the underlying immunotoxic mechanisms of AgNPs on the leukocytes of cetaceans may be different from those of humans. Considering the differences of cytotoxicity and immunotoxicity caused by AgNPs between the leukocytes of humans and cetaceans, it is suggested that AgNPs can cause different effects in different animal species in the ecosystem. To comprehensively evaluate the negative impacts of AgNPs on the biosphere, it is crucial to conduct more research to accumulate data on the biological effects of AgNPs in different animal species. However, the differences of AgNP-induced toxicity in different cells/animals may be also associated with the different state (such as coating, sizes, and the intracellular Ag^+^ ions release) of the AgNPs used in the *in vitro*/*in vivo* models. In addition, a recent study found that AgNPs could be generated from AgNO_3_ in culture medium, and the toxic effects induced by Ag^+^ ions and/or AgNPs may not be completely separated by using AgNO_3_ as a reference for *in vitro* models with AgNPs^52^. Therefore, further investigations to determine the underlying cytotoxic and immunotoxic mechanisms of AgNPs on the leukocytes of cetaceans with comprehensive AgNPs characterization and a suitable reference of Ag^+^ ions are warranted.

## Methods

All the experiments were performed in accordance with international guidelines^53,54^ and the manual for exposure control of nanoparticles in nanotechnology laboratories, which is published by the Institute of Labor, Occupational Safety and Health, Ministry of Labor, Executive Yuan, Taiwan. All reagents were purchased from Sigma-Aldrich (MO, USA) unless otherwise indicated.

### AgNPs characterization

C-AgNP_20_ (Pelco^®^ citrate Biopure™ silver) was purchased from Ted Pella (CA, USA). The AgNPs had been extensively washed without centrifugation to remove trace elements of the supernatant (<0.000001% of trace elements). TEM for determining surface area and size/shape distributions, UV–visible spectroscopy for measuring the optical properties, DLS for determining the particle hydrodynamic diameter and zeta potential were performed by the manufacturer and previous studies^29,30^. The endotoxin level of C-AgNP_20_ suspension was examined by ToxinSensor™ Single Test Kit (GenScript, NJ, USA) and was lower than or equal to 0.015 EU/ml. For characterization, the C-AgNP_20_ obtained from the manufacturer were suspended in complete RPMI-1640 medium at a concentration of 50 μg/ml, and then examined using a JEM-1400 TEM (JEOL, Japan). Briefly, the C-AgNP_20_ with complete RPMI-1640 was dropped on the TEM grids, stood for 30 min, and then dried with tissue paper (placed on the border of the grid to absorb the liquid). The size distribution and zeta potential were determined by dynamic light scattering (DLS) through Zetasizer Nano-ZS (Malvern Instruments Inc., MA, USA). Because 1) the relatively high recommended concentration (>100 μg/ml for) of nanoparticles for DLS, and 2) the difficulty on performing DLS in cell culture medium, only 100 and 500 μg/ml C-AgNP_20_ in 2 mM citrate buffer (pH 7.4) were measured through Zetasizer Nano-ZS in the present study^55^. Measurements were performed in triplicate and shown as means ± SD. The C-AgNP_20_ were diluted to 1, 10, 100, and 500 ug/ml with 2 mM citrate buffer and instantly used for subsequent experiments. Two mM citrate buffer was used as a vehicle control (0 μg/ml C-AgNP_20_).

### Blood sample collection

Voluntary blood samples were collected from captive dolphins in accordance with international guidelines, and the protocol had been reviewed and approved by the Council of Agriculture of Taiwan (Approval number 1051700175). Samples from six bottlenose dolphins (*Tursiops truncatus*) in Farglory Ocean Park were obtained on a monthly basis from 2015 to 2017. Blood samples were obtained from clinically healthy dolphins with confirmation by history, physical examination, complete blood count, and biochemistry. Forty millilitres of heparin-anticoagulated whole blood were collected, stored, and shipped at 4 °C within 8 hours for subsequent experiments.

### Blood sample preparation for subsequent experiments

For cytomorphological examinations, cytotoxicity assays, the detection of ROS production of cPMNs, and functional activity assays of cPBMCs, cetacean peripheral blood leukocytes (cPBLs) were isolated by a slow spin method and a density gradient centrifugation method with minor modifications^56^. The isolated cPBLs were resuspended in RPMI-1640 (Gibco) with 10% ethylenediaminetetraacetic acid (EDTA) and subsequently used in the isolation of cPBMCs by density gradient centrifugation method. After centrifugation at 1200 ×g for 30 min at 20 °C, the cPBMCs were collected from the cell layer between the RPMI-1640 (Gibco) and Ficoll-Paque PLUS (GE Healthcare, Uppsala, Sweden), washed with RPMI-1640 twice (if the cells were utilized in proliferative activity assay, the cells were washed with PBS once, and the procedures in the section of “functional activity assay of PBMCs” were followed), and resuspended to a final concentration of 1 × 10^7^ cells/ml in complete RPMI-1640 medium. The bottom sediment was collected and erythrocytes were lysed (Keogh *et al*. 2011). The cPMNs were washed and resuspended to a final concentration of 1 × 10^7^ cells/ml in complete RPMI-1640 medium. The cell viability of cPMNs and cPBMCs was determined by the trypan blue exclusion method using a hemacytometer, and the cell purity based on the cell size (forward-scattered light; FSC) and inner complexity (side-scattered light; SSC) of cPMNs and cPBMCs were determined by FACScalibur flow cytometry (BD, CA, USA). The cPMNs with higher than 90% viability and 90% purity and the cPBMCs with higher than 90% viability and 80% purity were used in this study. The isolated cPMNs and cPBMCs were kept on ice until utilized in subsequent experiments. For evaluating the effects of C-AgNP_20_ on the functional activity of cPMNs, the white blood cell (WBC) count of the whole blood sample was determined by VetAutoread Hematology Analyzer (IDEXX Laboratories, ME, USA), and the concentration of WBC of the whole blood sample was adjusted to a final concentration of 5.56 × 10^6^ cells/ml.

### Cytomorphological examinations and AMG silver visualization

Freshly isolated cPMNs and cPBMCs (1 × 10^6^ cells/ml in complete RPMI-1640 medium) were exposed to C-AgNP_20_ at concentrations of 0 and 50 μg/ml in duplicate. After 4 h of culture in an atmosphere of 5% CO_2_ at 37 °C, cells were cytocentrifuged (28 ×g; 10 min). One of the cytology slides was stained with Liu’s stain (Tonyar Biotech, Taipei, Taiwan) according to the manufacturer’s instructions. Another cytology slide was fixed with 100% methanol for 10 min at −20 °C, stained by silver enhancement method^15,57,58^, and counterstained with hematoxylin for 30 sec. AMG positive signals included golden yellow to black dots to an amorphous golden yellow substance. Freshly isolated cPMNs and cPBMCs (1 × 10^7^ cells/ml in complete RPMI-1640 medium) were exposed to C-AgNP_20_ at concentrations of 0 and 50 μg/m for 4 h, fixed with glutaraldehyde (2.5%), and examined using a JEM-1400 TEM (JEOL).

### Cytotoxicity assays of cPMNs and cPBMCs

The cytotoxicity of C-AgNP_20_ in cPMNs and cPBMCs was evaluated by the Annexin V-FITC/PI Apoptosis Detection Kit (Strong Biotech, Taipei, Taiwan) according to the manufacturer’s instructions. Briefly, freshly isolated cPMNs and cPBMCs were seeded in 96-well plates at a density of 5 × 10^5^ cells/well and exposed to C-AgNP_20_ at concentrations of 0, 0.1, 1.0, 10, and 50 μg/ml. After 1, 4, and 16 h of culture, cells were collected and resuspended in binding buffer for further analysis by FACScalibur flow cytometry (BD). A total of 8,000 events/sample were acquired. The sub-lethal doses of C-AgNP_20_ for cPMNs and cPBMCs were determined and further used in the functional activity assays.

### Functional activity assays of cPMNs

The effects of C-AgNP_20_ on the phagocytosis and respiratory burst of cPMNs were separately evaluated by the previously established protocol with minor modifications^59^. Briefly, 200 μl of heparinized whole blood samples was seeded in 48-well plates at a density of 1 × 10^6^ cells/well with or without sub-lethal doses of C-AgNP_20_ in duplicate. After 1 and 3 h of culture, 40 μl PI labelled *Staphylococcus aureus* (PI-staph) and unlabeled *S*. *aureus* (U-staph) were added for evaluating phagocytosis and respiratory burst, respectively. The whole blood samples with PI-staph or U-staph were incubated for 30 min in a shaking incubator (200 rpm) at 37 °C. PI-staph and U-staph were added for the final ratio of 30:1 (bacteria to leukocyte) to all relevant tubes. Subsequently, 10 μl DPBS and 10 μl DCFH-DA were added to the whole blood samples with PI-staph and U-staph, respectively. Further incubation for 20 min in a shaking incubator (200 rpm) at 37 °C was performed. Subsequently, the erythrocytes were lysed, washed, and resuspended in 350 μl cold DPBS with 1% paraformaldyde^59^. The ROS production of cPMNs induced by C-AgNP_20_ without bacterial stimulation was determined according to previous studies^30,31^. Briefly, freshly-isolated cPMNs were seeded in 96-well plates at a density of 5 × 10^5^ cells/well with different concentrations of C-AgNP_20_ (0, 0.1, 1.0, 10, and 50 μg/ml) in duplicate. After 1 and 4 h of culture, cPMNs were collected, washed and resuspended in DPBS for flow-cytometric analysis.

### Functional activity assay of cPBMCs

To evaluate the effect of C-AgNP_20_ on the functional activity of cPBMCs, the cell viability of cPBMCs exposed to C-AgNP_20_ at concentrations of 0, 0.1, 1.0 and 10 μg/ml with or without 2 μg/ml Concanavalin A (Con A; from Canavalia exsiformis, Sigma-Alderich, MO, USA) after 60 h of culture was determined by PI staining method with flow-cytometric analysis^31^. The sub-lethal dose of C-AgNP_20_ for cPBMCs was used in the functional activity assay of cPBMCs. The effects of C-AgNP_20_ on the proliferative activity of cPBMCs with or without Con A (Sigma-Alderich) were evaluated by flow cytometry using the Vybrant CFDA SE cell Tracer Kit (Molecular Probes, Oregon, USA) according to the manufacturer’s instructions with minor modifications. Briefly, cPBMCs were isolated, washed, and then resuspended in 5 ml DPBS with 10 mM Carboxyfluorescein succinimidyl ester (CFDA-SE). The cPBMCs were seeded in 96-well plates at a density of 3 × 10^5^ cells/well, and exposed to sub-lethal doses of C-AgNP_20_ with or without 2 μg/ml Con A. After 60 h of culture, cells were collected, resuspended in 200 μl of Accutase (StemPro^®^ Accutase^®^, Thermo Fisher Scientific, MA, USA), and incubated for 20 min. The cells were gently resuspended and transferred into 5 ml centrifuge tubes with cold 150 μl DPBS for flow-cytometric analysis using Modfit LT 3.0 software (Verity Software House, ME, USA).

### Statistical analysis

All experiments were independently repeated twice in duplicate (N = 12), except the experiments of (1) cell viability of cPBMCs after 60 h of culture and (2) detection of ROS production of cPMNs (N = 6). In all experiments, the results from duplicates were averaged. Individual differences in the ROS production of cPMNs and the functional activity assays of cPMNs and cPBMCs were observed in our study. To compensate for the individual differences, the results at different concentrations of C-AgNP_20_ for each individual were calculated as percentages of the results of the control^60^. Our data were first checked by Shapiro-Wilk normality test and Brown-Forsythe test, and results indicated that the assumptions of normality and/or equal variance were violated. Therefore, Kruskal-Wallis Test (post hoc test: Dunn’s multiple comparison test) was subsequently performed on the data, and the results in each experiment were compared to the control. Exceptionally, the Mann-Whitney U-test was used to compare (1) the cytotoxicity caused by C-AgNP_20_ between cPMNs and cPBMCs, and (2) the proliferation index between cPBMCs with or without Con A. A p value <0.05 was considered statistically significant, and the analysis was performed in Prism (GraphPad Software, CA, USA). All data were plotted on box-plot graphics. The bar in the middle of the box represented the second quartile (median), and the bottom and top of the box described the first and third quartiles. The whiskers showed the 75th percentile plus 1.5 times IQR and 25th percentile minus 1.5 times IQR of all data, and any values that are greater than these are defined as outliers and plotted as individual points. Asterisks above the boxplots indicated statistically significant differences compared to the control of each experiment.

### Ethical approval

All procedures performed in this study involving animals were in accordance with international guidelines, and the protocol had been reviewed and approved by the Council of Agriculture of Taiwan (Approval number 1051700175).

## Electronic supplementary material

Supplementary Information
