# Supplementary material for: Immunotoxicity of Silver Nanoparticles (AgNPs) on the Leukocytes of Common Bottlenose Dolphins (Tursiops truncatus)
Source: Sci Rep. 2018 Apr 4;8:5593. doi: 10.1038/s41598-018-23737-0 (PMC5884781; doi:10.1038/s41598-018-23737-0)
Supplement: Supplementary file 1 — Supplementary Information [file 41598_2018_23737_MOESM1_ESM.pdf]

Immunotoxicity of Silver Nanoparticles (AgNPs) on the Leukocytes of Common Bottlenose Dolphins (*Tursiops truncatus*)

Wen-Ta Li[1], Hui-Wen Chang[1], Wei-Cheng Yang[2], Chieh Lo[3], Lei-Ya Wang[1], Victor Fei Pang[1], Meng-Hsien Chen[4], Chian-Ren Jeng[1]

[1] Graduate Institute of Molecular and Comparative Pathobiology, National Taiwan University, Taipei 10617, Taiwan

[2] College of Veterinary Medicine, National Chiayi University, Chiayi 60004, Taiwan

[3] Farglory Ocean Park, Hualien 97449, Taiwan

[4] Department of Oceanography and Asia-Pacific Ocean Research Center, National Sun Yat-sen University, Kaohsiung 80424, Taiwan

*Corresponding author: crjeng@ntu.edu.tw

Supplementary Table S1. The size distribution and zeta potential of the 20 nm citrate-AgNPs ($C\text{-}AgNP_{20}$).

Concentration (µg/ml)	100	500
Z-Average (nm)	26 ± 0	26 ± 0
Size (nm) (intensity)	30 ± 0 (100%)	29 ± 0 (100%)
Zeta potential (mV)	-38.97 ± 1.33	-44.2 ± 1.35
PdI	0.12 ± 0.00	0.11 ± 0.01

Results shown are means ± SD from three different lectures.

NPs were suspended in 2nM citrate buffer and measurements performed at room temperature.

PdI, Poly-dispersity Index.

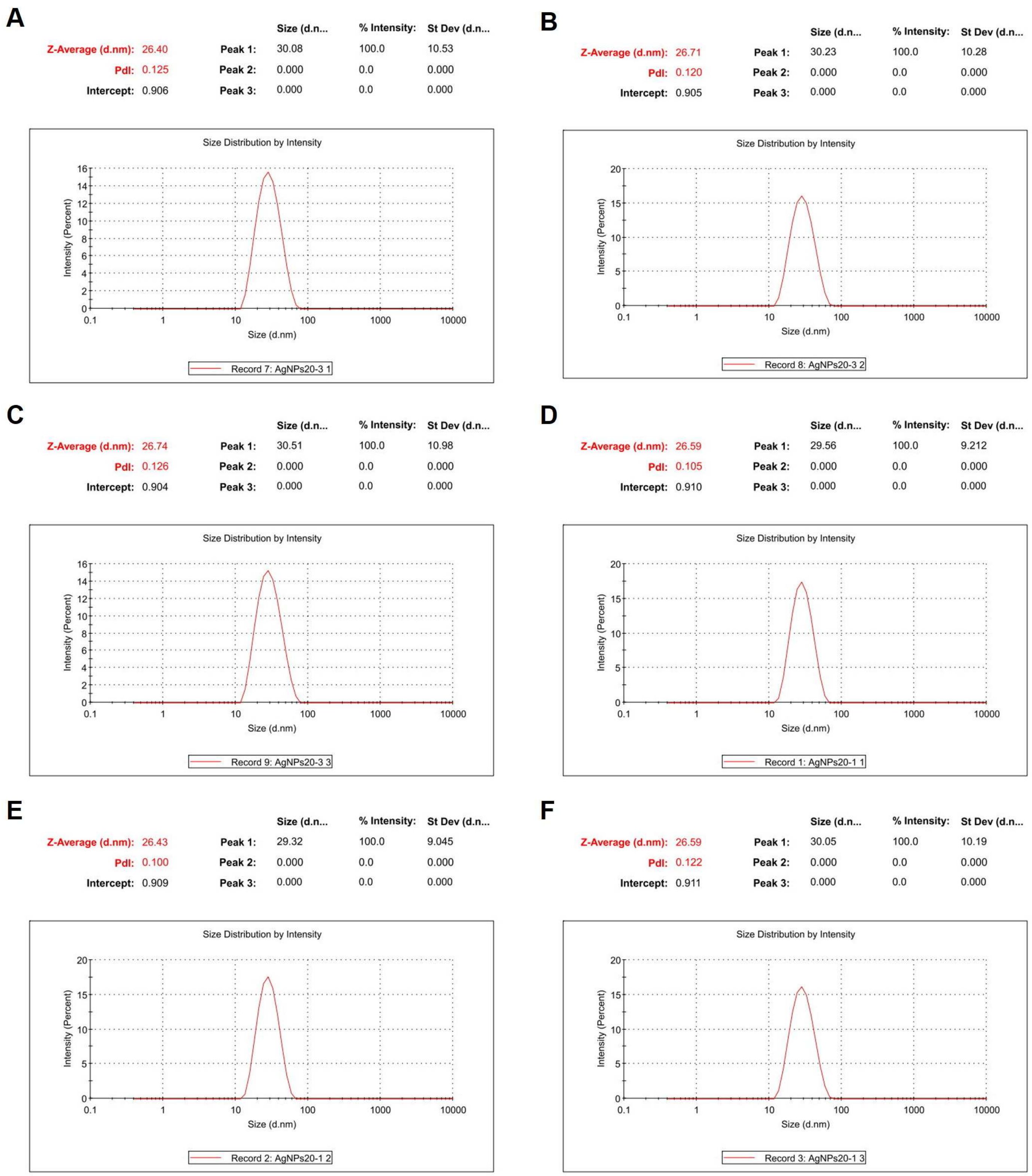

Supplementary Figure S1. The size distributions of C-AgNP$_{20}$ at 100 μg/ml (A to C) and 500 μg/ml (D to F).

A

			Mean (mV)	Area (%)	St Dev (mV)
Zeta Potential (mV):	-40.1	Peak 1:	-30.8	62.9	11.0
Zeta Deviation (mV):	26.4	Peak 2:	-56.2	14.4	5.35
Conductivity (mS/cm):	0.686	Peak 3:	-73.5	9.6	6.28

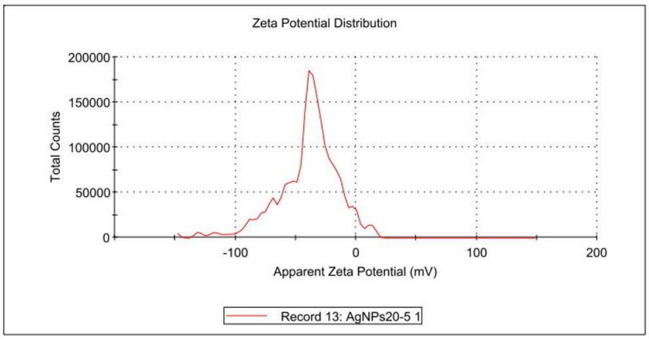

B

			Mean (mV)	Area (%)	St Dev (mV)
Zeta Potential (mV):	-37.1	Peak 1:	-49.8	41.3	10.8
Zeta Deviation (mV):	19.9	Peak 2:	-30.4	36.9	4.95
Conductivity (mS/cm):	0.709	Peak 3:	-12.6	19.3	6.81

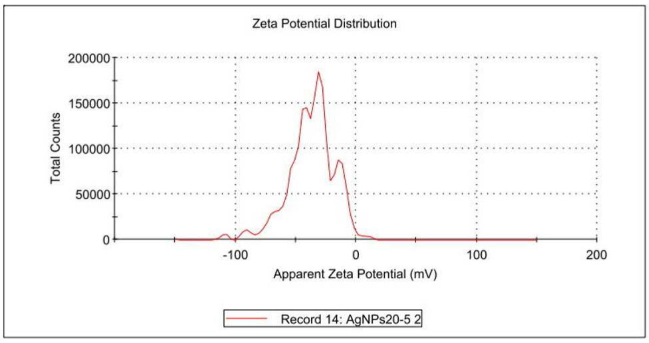

C

			Mean (mV)	Area (%)	St Dev (mV)
Zeta Potential (mV):	-39.7	Peak 1:	-37.8	59.4	9.17
Zeta Deviation (mV):	23.1	Peak 2:	-14.5	20.7	5.22
Conductivity (mS/cm):	0.722	Peak 3:	-74.6	8.2	6.24

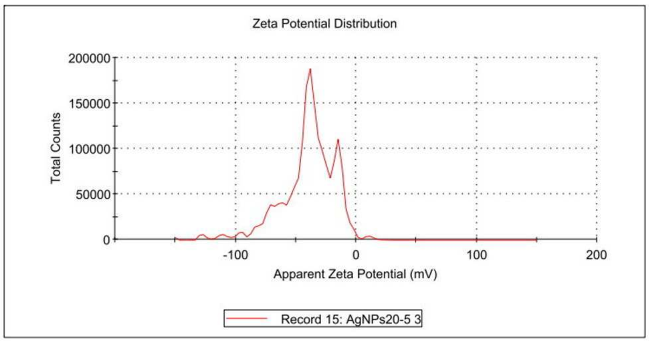

D

			Mean (mV)	Area (%)	St Dev (mV)
Zeta Potential (mV):	-43.1	Peak 1:	-39.5	88.6	12.4
Zeta Deviation (mV):	16.6	Peak 2:	-72.3	9.8	7.23
Conductivity (mS/cm):	0.690	Peak 3:	-97.8	1.5	5.15

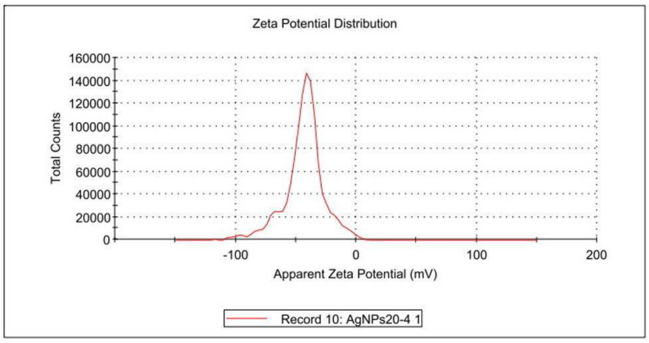

E

			Mean (mV)	Area (%)	St Dev (mV)
Zeta Potential (mV):	-43.4	Peak 1:	-37.2	69.8	10.6
Zeta Deviation (mV):	21.5	Peak 2:	-62.0	12.2	4.35
Conductivity (mS/cm):	0.714	Peak 3:	-81.7	12.0	9.65

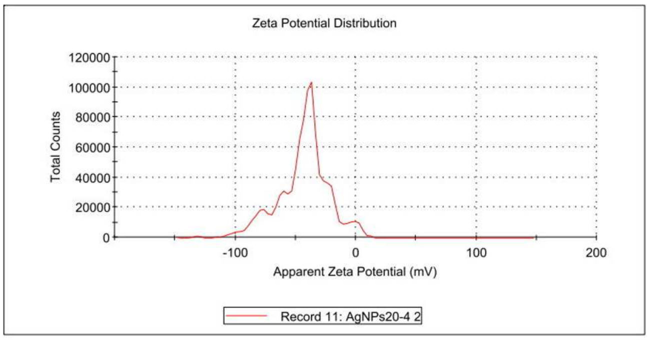

F

			Mean (mV)	Area (%)	St Dev (mV)
Zeta Potential (mV):	-46.1	Peak 1:	-32.7	34.5	7.11
Zeta Deviation (mV):	21.4	Peak 2:	-45.6	33.2	4.32
Conductivity (mS/cm):	0.725	Peak 3:	-61.2	19.9	6.73

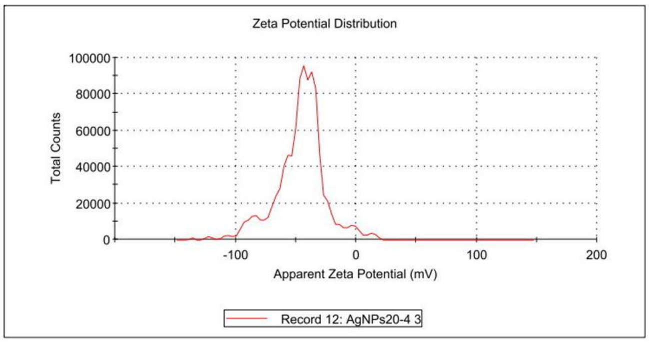

Supplementary Figure S2. The zeta potentials of C-$AgNP_{20}$ at 100 μg/ml (A to C) and 500 μg/ml (D to F).
